# Panhypopituitarism Secondary to Pituitary Abscess

**DOI:** 10.1016/j.aace.2024.08.004

**Published:** 2024-08-21

**Authors:** Samir S.E. Ahmed, Mona Vahidi Rad, Sydney Westphal

**Affiliations:** Division of Endocrinology and Metabolism, Mayo Clinic Arizona, Scottsdale, Arizona

### Case Presentation

A 59-year-old woman with a known history of cystic pituitary lesion for 2 years presented with a 3-week history of worsening daily headaches, loss of peripheral vision/double vision, and fatigability with increased thirst and frequent urination and no history of fever or night sweats. The laboratory results showed a prolactin level of 85 ng/mL (normal range, <25 ng/mL), thyroid-stimulating hormone level of 1.4 mIU/L (normal range, 0.4-4.2 mIU/L), free thyroxine level of 0.6 ng/dL (normal range, 0.9-1.7 ng/dL), follicle-stimulating hormone of 2.5 lU/L (normal range, 1.7-21 IU/L), luteinizing hormone level of <0.3 lU/L (normal range, 1.0-12.6 IU/L), and sodium level of 145 mg/dL (normal range, 135-145 mg/dL). Her pituitary magnetic resonance imaging showed significant enlargement of the cystic degeneration of the pituitary lesion with extension to the optic chiasm (Fig. *A*) in comparison to her previous magnetic resonance imaging on December 20, 2021 (Fig. *B*).

**Figure:**
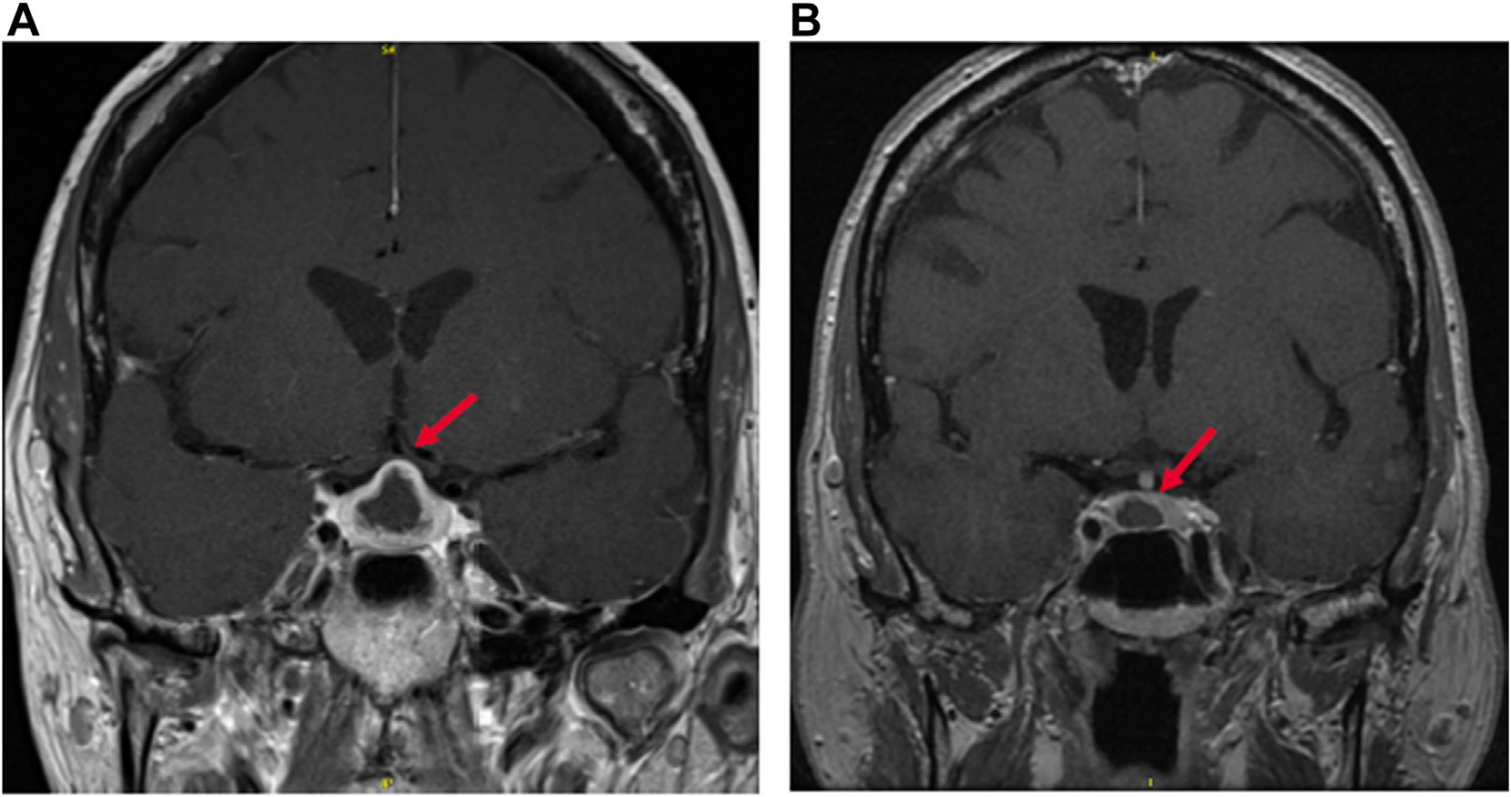

### What is the diagnosis?

#### Answer

Given the history and physical examination findings, which were concerning for panhypopituitarism, the differential diagnoses included pituitary apoplexy, Rathke cleft cyst, arachnoid cyst, pituitary abscess, cystic pituitary adenoma/cystic degeneration of a pituitary tumor, and epidermoid cyst. She underwent urgent transsphenoidal surgery and was found to have a pituitary abscess, which was drained surgically. Bacterial cultures grew *Enterococcus faecalis* and *Staphylococcus epidermidis*. She completed a total of 8 weeks of intravenous ceftriaxone and vancomycin plus oral metronidazole with no recurrence of the pituitary abscess. Postoperatively, she continued to require desmopressin, levothyroxine, and hydrocortisone therapy.

## Disclosure

The authors have no conflicts of interest to disclose.

